# Transforming long-term post-acute care for the aging population through home infusion therapy in China: an assessment of need and demand (Part 1)

**DOI:** 10.3389/fpubh.2026.1761871

**Published:** 2026-03-04

**Authors:** Wei Zuo, Zi Yin Zhou, Dylan H. Do, Austin-Phong Nguyen, Connie Vo, Tu Tran, Dan Mei, Shaohong Wang, Shan Li, Jianchun Yu, Hong Yang, Wei Chen, Bin Zhao, Luke Hoang, Michelle T. Le, Franco W. T. Cheng, Iris J. Chang, Jennifer Le

**Affiliations:** 1 Department of Pharmacy, Peking Union Medical College Hospital, Chinese Academy of Medical Sciences, Beijing, China; 2 Skaggs School of Pharmacy and Pharmaceutical Sciences, University of California, San Diego, La Jolla, CA, United States; 3 Dartmouth College, Hanover, NH, United States; 4 Tylan Health, El Monte, CA, United States; 5 Comfort Home Health & Hospice Care Inc., Long Beach, CA, United States; 6 Huntington Hospital, Pasadena, CA, United States; 7 Department of Medicine Intensive Care Unit, Peking Union Medical College Hospital, Chinese Academy of Medical Sciences, Beijing, China; 8 Department of General Surgery, Peking Union Medical College Hospital, Chinese Academy of Medical Sciences, Beijing, China; 9 Department of Gastroenterology, Peking Union Medical College Hospital, Chinese Academy of Medical Sciences, Beijing, China; 10 Department of Clinical Nutrition, Peking Union Medical College Hospital, Chinese Academy of Medical Sciences, Beijing, China; 11 Department of Pharmacology and Pharmacy, The University of Hong Kong, Hong Kong, China; 12 The Practising Pharmacists Association of Hong Kong, Hong Kong, China; 13 Center on International Pharmacy Education and Research, University of California, San Diego, La Jolla, CA, United States

**Keywords:** aging population, home health care, home infusion, outpatient parenteral antibiotic therapy, post-acute care

## Abstract

**Background:**

China’s healthcare system is confronting a rising burden of antimicrobial resistance, chronic complex diseases, and an aging population requiring long-term, post-acute care. As a value-based care model in the USA for over 50 years, home infusion therapy (HIT), combined with home health services (HHS), presents a sustainable alternative to prolonged hospitalization for intravenous medication administration. In this two-part narrative review, Part 1 provides a needs assessment describing the demographic, clinical, and public health factors driving the demand for HIT and HHS in China.

**Methods:**

We conducted a literature search up to January 2026 using MEDLINE, EMBASE, PubMed, Web of Science, and China National Knowledge Infrastructure.

**Results:**

In Part 1, we present the impact of the aging population and chronic conditions requiring prolonged infusion therapy (including cancer, malnutrition and infections like osteomyelitis, endocarditis, and bacteremia) on the rising antimicrobial resistance, hospital burden and healthcare expenditures. Through the patient-centric solution of HIT and HHS, patients can receive intravenous medications and nutrition in the comfort of their homes, enabling the continuity of care beyond the hospital. Services include outpatient parenteral antibiotic therapy, hospice and palliative care in patients with cancer, and management of nutritional needs through total parenteral nutrition. Under this care model, reimbursement is tied to success in improved patient outcomes and reduced hospital readmissions. A detailed reimbursement and cost-effectiveness considerations are addressed in Part 2 of this review.

**Conclusion:**

With proper infrastructure development and reimbursement mechanisms that align payment with value, HIT and HHS could mitigate antimicrobial resistance, and transform and sustain affordable care delivery in China, especially for older adults and those with chronic conditions.

Part 2 is available at https://doi.org/10.3389/fpubh.2026.1761870.

## Background

Antimicrobial resistance (AMR) in hospitals across China remains a major public health crisis, with high rates of multidrug-resistant organisms (MDROs) such as carbapenem-resistant *Enterobacteriaceae*, *Acinetobacter baumannii*, and *Klebsiella pneumoniae*. In fact for the past decade, national surveillance data from China Antimicrobial Resistance Investigation Net and China Antimicrobial Resistance Surveillance System report that resistance to carbapenems, which are considered the most reliable, last-resort treatment for serious Gram-negative bacilli infections, is high especially among *Klebsiella pneumoniae* and *Acinetobacter baumannii* (1–3). In addition, MRSA also contributes substantially to the antibiotic resistance, with MRSA contributing to one of the highest attributed deaths in recent years (4). Hospital-acquired infections are often associated with these MDROs, especially in intensive care units, and related to bacteremia and pneumonia (4, 5).

One of the key factors for MDROs in Chinese hospitals is prolonged hospital stays (6). Multiple efforts to address resistance have been implemented throughout Chinese hospitals, including antimicrobial stewardship programs, surveillance, and government-led campaigns to promote rational antibiotic use (1, 7). However, the burden of MDROs remains high, particularly among the older population (4, 8). Furthermore, China’s ongoing diagnosis-related groups and diagnosis-intervention packet (DRG/DIP) payment reform incentivizes shorter lengths of stay and standardized resource use, which aligns with the core value of home infusion therapy (HIT): enabling clinically stable patients to complete (9, 10). While HIT is well-aligned with the direction of national payment reform, implementation will require the establishment of reimbursement mechanisms, bundle payments, and inpatient to outpatient linkage models to ensure financial feasibility.

Home infusion therapy with home health services (HHS) is a promising solution for China’s evolving healthcare landscape, particularly with the rising AMR coupled to an aging population and the need for affordable and sustainable healthcare. By enabling patients to receive intravenous treatments for their post-acute care at home under professional supervision, HIT promotes appropriate use of antimicrobial agents, minimizes unnecessary hospital exposure, decreases the risk of hospital-acquired infections (HAIs), and prevents the spread of AMR pathogens (9, 10). Furthermore, HIT reduces hospital stays by shifting care from hospitals to homes, decreasing hospital burden and inpatient healthcare costs.

As a sustainable value-based healthcare delivery model in the United States of America (USA) for over 50 years, HIT aligns with China’s public health goals to decrease overuse of broad-spectrum antibiotics and improve long-term disease management, especially in the older population (11–13). In the USA, HIT is categorized as an alternate-site, community-based care modality. When mapped to China’s three-tier hospital system, HIT appears to fit in the top tier for specialized, complex care like long-term antibiotics. The DIP/DRG reforms intend to drive a redistribution of patients from tertiary to secondary to primary hospitals, and ultimately to community- or home-based care. However, evidence shows that under DIP, tertiary and secondary hospitals attract patient volume from primary hospitals owing to medical service superiority and capabilities (14). As such, HIT provides a clinically appropriate destination for stable patients who do not require inpatient monitoring but needs completion of infusion therapy. A major advantage of HIT is its service to aging adults with chronic conditions who prefer healthcare in the convenience of their home or a long-term care facility, offering continuity of care without the financial and logistical burdens of hospitalization. China’s existing “home sickbed” and “Internet + Nursing Services” pilots have provided initial structure for limited HHS services with potential HIT integration. With proper regulatory support, workforce training, and integration into community health systems, HIT could become a cornerstone of China’s strategy to modernize and humanize its healthcare delivery.

With the focus on China, the objectives of our two-part narrative review were to: evaluate the current need and demand for HIT, including services to the aging population (Part 1); assess the structure of the HIT and HHS models (Part 1); and formulate recommendations for successful implementation of HIT, incorporating cost-savings, technological innovation, and accreditation and certification standards into existing infrastructure for home-based care in China (Part 2 as a separate review). The HIT combined with HHS model for post-acute, long-term care offers a transformative and sustainable solution in China for three primary reasons: decrease AMR, provide long-term care for the aging population, and improve healthcare affordability.

## Methods

We searched online databases, including MEDLINE, EMBASE, PubMed, Web of Science, and China National Knowledge Infrastructure for original research and review articles published from database inception to January 2026. The key words used were home infusion, home parenteral infusion, outpatient infusion, outpatient parenteral therapy, outpatient parenteral antibiotic therapy, injection at home, home health, home care, and infusion center.

Our inclusion criteria were articles written in English or Chinese that examined HIT pertaining to safety, effectiveness, quality of life, patient-centered outcomes, satisfaction, and cost. Articles that were duplicates or published in languages other than English or Chinese were excluded. Part 1 was intentionally designed as a narrative review, as the objective was to synthesize heterogenous evidence from epidemiologic trends, policy documents, demographic data, and operational characteristics of China’s health system. A narrative review framework was appropriate to enable integration of diverse data sources that cannot be meaningfully pooled through systematic review methods. Therefore, PRISMA procedures and systemic screening were not applied.

## Results

The growth of the older population accompanies a substantial escalation in healthcare spendings that surged from 458.66 in 2000 to 5259.83 billion CNY in 2017 (15). Meanwhile, the dependency ratio of China’s older population doubled, from 11.9% in 2010 to 20.8% in 2021, resulting in a significant decline in the labor workforce from 8.4 working-age persons per old person to 4.8 (16). Under the Confucius influence on China’s filial piety culture, older adults rely on informal care provided by their children and family (17). Historically, adult children and family members serve as the dominant long-term caregivers, revealing the preference of older people in China to live and be cared for at home (18). The current landscape of older population care continues a shift to provide formal long-term services for the older population (19). A dramatic decline in the number of living children for each older person in China brings substantial challenges for both family-based care and social care (13). Notably, China’s 4–2-1 family structure (four grandparents, two parents, and one child) has intensified caregiving pressure on families. As a result of the one-child policy, one adult (child) is now responsible for supporting multiple aging family and relatives, often maintaining a full-time employment which significantly limits the household’s capacity to provide complex day-to-day medical care.

In addition to older population care, the escalating burden of chronic and complex conditions (including cancer, certain infections requiring prolonged treatment, nutrition, hospice, and palliative care) requires a cost-effective healthcare delivery model, like HIT and HHS, to support these patients requiring long-term treatment. These populations often require sustained, high-quality medical attention that is accessible and culturally sensitive, meeting patients’ preference for home treatment with comfort and offering convenience for caregivers and family members. In the sections that follow, we explored the current urgent need and demand for HIT and HHS in China.

### Aging population

Declining fertility and rising life expectancy are major contributors to the aging population in China (13). In 1950–1955, the total fertility rate in China was 6.1 but dropped to 1.3 by 2020 (13). In 2021, the total number of births was a record low of 10.62 million, while life expectancy at birth went from 44 years to 78.20 years (13). According to China’s Seventh National Census, 264 million adults are aged ≥60 years or older, and 190.64 million Chinese residents are ≥65 years old (16, 20, 21). In Hong Kong, a Special Administrative Region in China, 1.45 million people aged 65 years and over, accounting for 19.6% of the regional population (22). In response, the government has implemented a policy of aging in place, encouraging the private sector to offer services to the older population (22).

In 2019, the mortality rate of chronic non-communicable diseases (NCD) among the 60 years and above was 31,238 per 100,000 individuals (23). Non-communicable diseases accounted for 93.9% of all deaths in that age group (23). The top three diseases among the older population in China that contributed to the highest Disability-Adjusted Life Years (DALYs) were stroke (19.202 million DALYs), ischemic heart disease (13.895 million DALYs), and chronic obstructive pulmonary disease (9.453 million DALYs) (23). These three conditions made up 41% of total disease burden among the older population, demanding integrative NCDs management and shift post-acute inpatient care to community settings (23).

A promising solution is HIT combined with HHS, which has successfully played a critical role in addressing the long-term health care needs of older patients in some countries outside of China. The focus on long-term, post-acute care is aligned with the services that HIT and HHS deliver ranging from medical, nursing, and rehabilitative care for patients after an acute illness that requires traditional hospitalization (21). Notably, HIT allows patients to receive intravenous medications and nutrition in the comfort of their homes, reducing the burden on hospitals while improving patient outcomes. It enables continuity of care beyond the hospital as services include intravenous treatments for infections like endocarditis through outpatient parenteral antibiotic therapy (OPAT), hospice and palliative care in patients with cancer, and management of nutritional needs through total parenteral nutrition (TPN).

### Infections

Certain infections, including osteomyelitis and endocarditis, require prolonged intravenous antibiotic therapy, often extending over weeks to months. Vertebral osteomyelitis is increasing due to improved diagnostics and aging populations (24). The estimated incidence of vertebral osteomyelitis is 2.4 cases per 100,000 individuals, and a higher prevalence was observed in the older population aged 70 and above, with the incidence rate of 6.5 cases per 100,000 individuals (25). In China, *Mycobacterium tuberculosis* was reported as the most common microorganism in vertebral osteomyelitis, followed by *Staphylococcus aureus* and Brucella (25).

Hematogenous osteomyelitis carriers an incidence of ranging from 1 to 13 per 100,000 (26). The total financial burden for hospitalized patients with hematogenous osteomyelitis was 8,832,910 CNY for 259 patients hospitalized during the 10-year study period from 2011 to 2020 (27). The median hospital cost was 25,754 CNY per patient (range 11,502–47,661 CNY) (27). Among the care costs, drug cost accounted for the largest proportion (36%), followed by materials (24%), anesthesia and surgery (17%), diagnosis (16%), and comprehensive care (5%) (27).

Infective endocarditis (IE) remains relatively rare but serious in China, with an estimated incidence of 13.8 cases per 100,000 population annually as of 2019 (28). In a cohort of 961 IE cases in China, the overall in-hospital mortality rate increased from 7.7 to 26.4% with a higher rate of 38% in the older population (28). Among patients with IE, advanced age, higher Charlson Comorbidity Index, impaired renal function, healthcare-associated infection origin, and infection caused by methicillin-resistant *Staphylococcus aureus* (MRSA) were associated with increased mortality (29). The increased mortality associated with healthcare-associated endocarditis may likely reflect the burden of comorbidities and rising MRSA prevalence in hospitalized patients. As such, minimizing prolonged hospitalization to mitigate healthcare-associated endocarditis caused by MRSA is prudent to optimize patient outcomes.

Patients with these infections currently in China require hospitalization even for extended treatment duration since access to intravenous medications are unavailable outside the hospital. With hospital overcrowding and AMR presenting as urgent issues in China, HIT offers a safe and cost-effective alternative to inpatient treatment for these infections in otherwise stable patients who can complete their antibiotic course safely at home (30). The AMR-related benefits of HIT depend on strong antimicrobial stewardship infrastructure, including remote monitoring systems and treatment-duration oversight (31, 32). By aligning OPAT guidelines within HIT for patients with osteomyelitis, endocarditis and potentially other infections requiring extended treatment duration, China can improve treatment adherence, reduce healthcare costs, and enhance patient quality of life. Notably, OPAT expands hospital capacity through early discharge and admission avoidance, saving bed days, and improving patient flow as it shifts clinically stable patients out of high-risk inpatient environments (33). In recent decades, OPAT has been increasingly utilized internationally in managing these infections (34). Data from a 2022 meta-analysis of 9 studies and a total 1,116 patients with IE treated using OPAT reported occurrences of morbidities and mortality during treatment and follow-up period after discharge, including adverse events (26%), readmission during OPAT treatment (18%), infection relapse (3%) and mortality (4%) (35). Notably to date, there is a lack of studies specific to China. Most of the studies were conducted in high-income countries with robust OPAT infrastructure – including trained multidisciplinary teams, reliable home care support, and systemic patient monitoring. The importance of embedding HIT within China’s existing “Internet + Nursing Services” frameworks help ensure that OPAT delivery includes standardized treatment plans, laboratory monitoring, and early detection of complications. When implemented with these safeguards, HIT-supported OPAT has the potential to improve the continuity of care, relieve the burden of hospitals, and reduce unnecessary MDROs exposure.

### Cancer

In 2022, over 2.8 million new cancer cases and over 1.9 million cancer-related deaths were among Chinese adults aged 60 (36). The age-standardized incidence rate for all cancers was 1211.8 per 100,000 population, with Chinese adults aged 60 and older facing a heavy burden of lung and digestive cancers (36). Given the rapid aging population and limited healthcare resources, tailored, evidence-based strategies are urgently needed to improve cancer prevention and control in China (36).

In China, chemotherapy for patients with cancer has been administered on an outpatient basis within hospital settings, especially in urban areas such as Wuhan (37). The implementation of the outpatient mutual-aid policy has significantly shifted cancer delivery from hospitalization to outpatient treatment (37). This policy allows outpatient services such as chemotherapy and radiotherapy to be reimbursed at similar rates to inpatient care (50–68%), effectively making outpatient oncology treatment more accessible (37). As a result, oncology patients under this policy had shorter inpatient stays by 1.2 days and lowered hospitalization costs by 5% (37). The evolving outpatient cancer care model provides a relevant and timely framework for implementing HIT and HHS in China. Moreover, the 2024 Chinese expert consensus on patient-centered cancer care highlights home-based medical services, community infusion centers, and internet-enabled supportive care as core components of modern oncology delivery in China (38). These priorities directly align with the proposed HIT and HHS model, particularly combining administration of chemotherapy at infusion centers and nutrition or palliative care at home.

### Nutrition

Malnutrition is prevalent in Chinese older population, impacting their dependency on physical function and activities of daily living (15, 39). The prevalence of malnutrition among older Chinese individuals with physical functional dependency was 17.9% (40). Malnutrition poses a great burden on healthcare resources through increased hospitalization, infection, mortality, and costs for the care of the older population (39). In a well-structured HIT program, total parenteral nutrition (TPN) can be administered safely at home under clinical supervision, supporting recovery, and improving quality of life. Nutritional support is a medical treatment for malnutrition that encompasses enteral and parenteral nutrition (39). There is a strong recommendation for TPN use in older patients with severe gastrointestinal dysfunction, or other contraindications to the use of enteral nutrition since they cannot tolerate or fully absorb nutrients (41). For HIT-compounded TPN, it is important to ensure temperature-controlled transport from the pharmacy to the patient’s home. Refrigerated courier services are generally used for scheduled home delivery (often weekly or biweekly). Real-time tracking and temperature monitoring for TPN or other drugs may be prudent. Any HIT model should include contracted medical-waste collection pathways to ensure proper disposal and environmental safety. Given that TPN is often required long-term, it necessitates careful management that can be effectively provided in a home setting for clinically-stable patients.

Hospitalization is prudent to initiate TPN until a consistent TPN regimen is identified. While most patients require only short-term TPN for use, some may require prolonged TPN. When patients require prolonged TPN, home-based TPN for clinically stable patients offers a safe and effective alternative for patients to maintain independence and quality of life while managing their medical needs in a familiar environment. With proper training, patients and caregivers can safely administer TPN at home, care for central venous catheters, and monitor for potential complications (such as infections or liver disease as reported in older patients on long-term TPN). Home-based TPN also reduces healthcare costs and minimizes the risk of HAIs (39). However, successful implementation depends on a strong support system, and regular follow-up with a multidisciplinary care team. Notably, HIT pharmacies provide the structural framework to provide TPN product compounding under sterile environment outside of hospitals to the home or long-term care facilities. Furthermore, special formulations of enteral nutrition are usually accessed via HIT when outpatient care is needed.

### Hospice and palliative care

Most hospice patients prefer to spend their final stages of life at home due to limited medical resources and the traditional cultural concept of life closure; the cultural preference for passing away at home (42). Since 2017, China has referred to end-of-life care and palliative care collectively as hospice care (12, 43). With the increasing aging of the population and the increasing incidence of chronic diseases in China, home hospice care services can respect the desire of the older population with chronic diseases to receive care and to die at home (43). As more than one-fifth of the population of China are now aged 65 years old or older, it is vital to ensure the well-being of chronically ill older adults at the end of life (44).

Hospice and palliative care (HPC) are vital for patients with cancer nearing the end of life. In a large multicenter retrospective study of 3,350 Chinese patients with advanced solid malignancies, 11.6% received chemotherapy within the last month of life, and over 5% received chemotherapy within the last 2 weeks (45). Receiving chemotherapy so close to death were associated with shorter overall survival, higher rates of intensive care unit admissions, and a greater likelihood of in-hospital death. These findings underscore the need for earlier integration of palliative care and shift away from aggressive treatments near the end of life (45).

Over the past decade, China has made notable advancements in HPC in response to the aging population and rising cancer burden. Government-led initiates such as “Health China 2030” and multi-tier pilot programs have expanded HPC access across urban and rural settings, provided standardized care guidelines, increased opioid availability for pain control, and trained over a thousand specialized palliative care nurses (46). Ongoing challenges include resource distribution, timely transition to HPC, insufficient training in grassroots institutions (e.g., hospitals and hospice care centers), and a need to shift clinician and patient mindsets from curative intent to comfort-focused care (46).

The deeply rooted cultural norms equating hospital care with superior care reflect how many families associate home-based care with “giving up” and patients may prefer hospital settings even when home care may be better support, comfort, dignity, and family presence. Currently, HPC represents a key context where both hospital-centric cultural perception of superior inpatient treatment and the caregiving constraints of the 4–2-1 family structure intersect, underscoring the need for professionally delivered HIT services (47). Despite clear benefits, HPC continues to be underutilized, even in countries with existing HIT infrastructure (48). Barriers include misconceptions about their purpose, difficulty accepting mortality, and systemic challenges in access and coordination. These barriers need to be addressed to ensure that all patients with advanced cancer will receive high-quality, person-centered end-of-life care aligned with their values and goals.

### Affordability and sustainability

With the increasing burden of chronic complex diseases and aging populations worldwide, post-acute care models are under pressure to deliver high-quality care while reducing costs. Nursing homes remain essential for patients requiring intensive care after hospitalization, but many seniors prefer HHS. In this context, HIT combined with HHS has emerged as a cost-effective and sustainable alternative to prolonged hospitalization for patients requiring long-term intravenous or subcutaneous therapies such as OPAT (for osteomyelitis and endocarditis), home TPN, and HPC (Table 1).

**Table 1:** Type and description of home health services.

Type of care	Description
Skilled nursing care	Administering medications and injections Wound care and dressing changes Monitoring vital signs and health conditions Pain management Disease management (diabetes, COPD, heart failure) IV therapy and injections Catheter care and maintenance
Physical therapy	Rehabilitation after injury or surgery Strengthening exercises to improve mobility Training in the use of assistive devices (e.g., walkers, canes) Balance training and fall prevention Range of motion exercises
Occupational therapy	Help with activities of daily living (ADLs), such as bathing, dressing, and eating Recommendations for adaptive equipment to improve independence Home safety assessments Energy conservation techniques for chronic illness or disability
Speech therapy	Treatment for speech, language, or communication disorders Swallowing therapy for patients with dysphagia Cognitive rehabilitation for memory or thinking problems after stroke or injury
Medical social services	Counseling for emotional or social challenges related to health conditions Help with long-term care planning Assistance in accessing community resources (financial, housing, etc.) Support in adjusting to lifestyle changes due to illness or disability
Home health aide services	Assistance with personal care (bathing, grooming, dressing) Help with mobility (transferring, walking) Light housekeeping, laundry, and meal preparation Basic companionship and socialization
Personal care services	Help with daily activities such as feeding, toileting, and grooming Companionship services Non-medical support for routine personal tasks
Companionship services	Social interaction and emotional support Accompanying patients to appointments Assistance with hobbies, reading, and other leisure activities
Palliative care	Symptom management for serious or chronic illnesses Emotional and spiritual support Assistance with decision-making for care planning Coordination with other healthcare professionals for comprehensive care
Hospice care	End-of-life care focused on comfort, not cure Pain management and symptom relief for terminally ill patients Emotional and spiritual support for patients and families Bereavement support for families after a patient’s death
Nutritional counseling	Meal planning and dietary advice for chronic conditions (e.g., diabetes, heart disease) Monitoring nutritional intake Specialized diets for conditions like kidney disease, cancer, or gastrointestinal disorders
Respiratory therapy	Oxygen therapy management Ventilator care and training Nebulizer treatments Monitoring and treating respiratory conditions (COPD, asthma)
Pharmacy services	Delivery of medications to the home Instruction on proper medication use Monitoring drug interactions and side effects
Infusion therapy	Intravenous (IV) administration of medications, fluids, and nutrition Treatment for conditions like infections, cancer, dehydration, and immune disorders
Wound care management	Complex wound care, including pressure sores, surgical wounds, and diabetic ulcers Use of advanced dressings and wound care treatments Monitoring for infection and complications
Post-surgical care	Follow-up care after surgery, including wound management Monitoring for complications, infections, or adverse reactions Pain management and mobility support
Dementia and Alzheimer’s care	Specialized care for individuals with cognitive impairments Assistance with memory-related challenges and behavioral management Structured daily activities to support cognitive function
Home medical equipment and supply services	Delivery and setup of equipment like hospital beds, wheelchairs, or oxygen Instruction on the proper use of medical devices Regular maintenance and replacement of equipment
Chronic disease management	Ongoing monitoring of chronic conditions like diabetes, hypertension, and heart disease Education on managing symptoms and lifestyle changes Coordination of care with other healthcare providers
Rehabilitation services	Services designed to help patients regain physical, mental, or cognitive abilities lost due to injury, surgery, or illness
Remote patient monitoring	Use of technology to monitor patients’ vital signs and symptoms remotely Provides real-time data to healthcare providers for adjustments to treatment plans These services are tailored to each patient’s individual health needs, and the goal is to maintain or improve the patient’s quality of life while allowing them to remain in their home

In addition to the increase in chronic complex diseases and aging populations, China also faces significant hospital burden and rising healthcare costs. China’s establishment of long-term care insurance in 2016 further reflects the need for a robust and sustainable healthcare and insurance system to effectively meet the evolving treatment needs for chronic and complex medical conditions, especially for long-term and post-acute care services (20). Furthermore, China’s Central Committee approved the Healthy China 2030 plan in the effort to strengthen effective management for older individuals with cognitive impairment (49). Home-based care remains to be a preferred choice for many older adults (49).

The sustainability of a HIT model depends on adequate nursing capacity, a challenge given that China has approximately 3.7 nurses per 1,000 population, which is below the 5 per 1,000 level established by the Organization for Economic Co-operation and Development. A recent study revealed that China faces a significant shortage of experienced and highly trained nurses (50). Notably, HIT may address the nursing shortage by decentralizing care and reallocating specialized labor away from overburdened hospital into a patient’s home. By shifting clinically stable patients to the home, health systems can preserve high-acuity hospital beds and the corresponding bedside nursing staff for emergencies.

In the sections that follow, we presented HIT and HHS within the framework of value-based care healthcare delivery, incorporating experience from the USA with existing successful implementation for over 50 years (11, 51). These programs provide significant value-based benefits, including improved clinical outcomes, continuity of care beyond hospitalization, enhanced patient autonomy and quality of life, reduced hospital readmissions, and safer home-based management of complex therapies. Economically, HIT and HHS reduce healthcare costs through shorter hospital stays, lower inpatient treatment expenses, and decreased risk of healthcare-associated infections.

### Value-based care in the United States

Value-based care is a healthcare framework that emphasizes patient-centric care by improving clinical outcomes while enhancing patient experience with safety and convenience. As a specialized component of home healthcare with value-based care, HIT with HHS services involve the intravenous or subcutaneous administration of drugs or biologicals to an individual at home, or an outpatient facility called infusion center. The components needed to perform HIT include the drug (e.g., antibiotics, immune globulin), equipment (e.g., a pump), and supplies (e.g., tubing and catheters). Value-based care models like HIT and HHS enhance the effectiveness and appeal of HHS for seniors and others with long-term post-acute care needs (51). Healthcare systems and payers increasingly recognize HIT’s role in reducing hospital burden and overall healthcare costs (52–54).

To enhance care quality and efficiency within value-based care, HIT and HHS were implemented in the USA in the 1970’s with reimbursement by Medicare shortly after the program’s initiation (11). Medicare is the federal public health insurance program and largest single payor for healthcare in the USA, especially for people 65 years or older and those with chronic diseases. While initial reimbursement for HIT and HHS was limited, efforts to extend coverage transpired in 1980. Notably in 2016, the Center for Medicare and Medicaid Innovation launched the Home Health Value-Based Purchasing Model (55). This initiative incentivized agencies within HHS and HIT to deliver higher-quality care by linking payments to performance. Furthermore, this model led to a 4.6% improvement in performance scores and saved an average of $141 million annually—while maintaining quality and safety of care delivery. It also reduced unplanned hospitalizations and stays at skilled nursing facilities, thereby lowering overall inpatient and other healthcare expenditures (55).

Building on this success, the reimbursement model for HIT and HHS was expanded in 2022 across the USA, and continues to reduce unnecessary emergency visits, improve patient outcomes, and reduce overall healthcare costs. Specifically, HIT programs that have demonstrated substantial cost savings compared with inpatient care are OPAT and home TPN. Multiple studies have reported that the total cost for HIT on average ranged from approximately $40,000 to over $80,000 per patient (56). On a per-treatment basis, HIT was typically $1,900 to $3,000 less expensive than comparable medical setting infusions (57). One modeling study using Medicare data, developed in 1998, estimated that OPAT implementation nationwide could result in a cumulative savings of nearly $3 billion in 2023 dollars over 5 years (56). A detailed analysis of the cost-savings was presented in Part 2 of this narrative review.

Currently, federal Medicare, state Medicaid, and most private insurance programs provide some level of reimbursement for HIT and HHS services in the USA (58). However, the reimbursement process for HIT and HHS is complex, and requires thorough documentation, insurance verification, and prior authorization. Furthermore, the exact reimbursement rate and patient out-of-pocket cost varies by type of public and private insurance, forcing patients to continue therapy at a skilled nursing facility, or infusion centers rather than home to minimize patient out-of-pocket cost.

The COVID-19 pandemic accelerated the shift toward home-based care, including HIT, offering a safer alternative to hospitalization amid capacity limits and infection risks. The availability of HIT enabled patients—particularly those with chronic or immunocompromising conditions—to continue essential intravenous treatments at home (59). The administration modalities such as intravenous push, elastomeric pumps, gravity infusions, ambulatory pumps, and stationary pumps enabled a safe, effective, and patient-centered delivery of antimicrobials, antivirals, and other therapies across home and outpatient settings (59). In particular, OPAT was rapidly adapted and enhanced with telemedicine to provide antibiotic treatment without requiring hospitalization during the pandemic (60). Rising demand during the pandemic spurred innovations such as telehealth support, remote monitoring, and caregiver training. The current condition highlights the importance of continued reform in the USA to continue to enhance reimbursement for HIT and HHS to ensure its sustainability (61).

Under the HIT and HHS value-based care model, reimbursement is tied to success in improved patient outcomes, reduced hospital readmissions, and cost efficiency. This model incentivizes providers to deliver high-quality, cost-effective care by aligning payment with value, which is essential for those with long-term, post-acute care needs. Establishing a proper and effective reimbursement structure is essential for the sustainability and accessibility of these programs. Without a robust reimbursement process, patients may face significant financial barriers, delays in care, or even inability to access necessary therapies, especially for the largest consumers of healthcare expenditures—older adults and those with chronic complex diseases (62).

### Policy implications of value-based care in China

The extensive USA experience with HIT and HHS within value-based care demonstrates that home-based infusion services is a safe and effective alternative to hospitalization while reducing overall healthcare cost. Long-term success is contingent upon reimbursement models, with payment linked to measurable quality indicators in patient outcomes reductions in emergency department visits and hospital readmissions. China’s adoption of HIT and HHS faces intersecting regulatory, reimbursement, technical, and cultural challenges despite decades of experience in the USA and Europe. A China-tailored model must align with national policies, integrate with clinical workflows, and account for the existing workforce. The most immediate barrier is the lack of regulatory infrastructure for accreditation, certification, and liability protection—mechanisms essential for managing provider risk and ensuring patient safety (63–65). Coupled with this, shortages of trained technical staff, particularly nurses specialized in homebased care, further complicate implementation (63–66).

Developing a sustainable HIT and HHS payment model also requires coordinated action among insurers, government agencies, healthcare providers, and patients. Existing reforms may offer strategic opportunities: the Zero Markup policy reduces reliance on drug revenue and shifts emphasis toward professional service value, while DRG/DIP–based bundled payments encourage hospitals to transition appropriate care to lower-cost settings such as HIT (67–70). To fully integrate home infusion therapy into China’s reimbursement framework, stakeholders must continue advancing payment mechanisms that support safe, efficient home-based treatment (67, 71).

Socioeconomic and cultural factors will shape acceptance and effectiveness. Specifically, HIT can improve quality of life by allowing patients to resume daily routines while remaining closely connected to healthcare professionals through 24/7 access, follow-up visits, and remote monitoring (63, 72). However, broader acceptance requires patient and family education, caregiver training, and the expansion of monitoring technologies (63–65, 73). Addressing disparities in medical resources, particularly between urban and rural areas, will also be essential (67, 71, 74).

## Conclusion

Home infusion with HHS offers a safe, effective, and economical alternative to inpatient hospital care. In China with the rising AMR, hospital burden and healthcare cost due to the aging population and need for long-term, post-acute healthcare delivery, HIT and HHS is a promising solution for affordability and sustainability. While international experience provides a valuable foundation, HIT’s adaptation to China will require phased, long-term investment in regulation, workforce development, technology, and payment reform to ensure feasibility and equitable access (67, 71). The fragmented health information technology infrastructure—with separate systems for clinical care and public health that are rarely interoperable—poses additional challenges for coordinating home-based services (67, 74).
